# Neutrophils in tumor- and inflammation-induced lymphangiogenesis

**DOI:** 10.7150/ijbs.103458

**Published:** 2025-02-26

**Authors:** Xinyu Li, Xiaoxin Liu, Haotian Wei, Yanyan Liu, Gang Xu

**Affiliations:** 1 Department of Nephrology, Tongji Hospital, Tongji Medical College, Huazhong University of Science and Technology, 430030 Wuhan, Hubei, P. R. China.; 2 Department of Nephrology, Liyuan Hospital, Tongji Medical College, Huazhong University of Science and Technology, 430077 Wuhan, Hubei, P. R. China.

**Keywords:** Neutrophils, Lymphangiogenesis, Lymphatic Endothelial Cells (LECs), Tumor, Inflammation, Therapy

## Abstract

Lymphangiogenesis is the formation of new lymphatic vessels from preexisting vessels and occurs during embryonic lymphatic development and under pathological conditions induced by internal or external stimuli. Emerging evidence suggests that neutrophils contribute to the construction and remodeling of new lymphatic vessels. Neutrophils migrate to lymph nodes through the lymphatic vessels or high endothelial venules, and neutrophil migration may depend on the phenotype of the neutrophil. The presence of unique neutrophil phenotypes in individuals with lymphangiogenesis has been reported. Neutrophils promote lymphangiogenesis mainly by secreting lymphotropic factors or increasing their bioavailability and by collaborating with various immune cells. Neutrophils mediate lymphangiogenesis and exert complex effects on tumors and inflammation. The selective inhibition of specific neutrophil and neutrophil lymphangiogenic molecules may provide a novel approach for the prevention and treatment of associated diseases.

## Introduction

Lymphatic capillaries are thin-walled vessels lined by a monolayer of lymphatic endothelial cells (LECs), which express specific markers, including vascular endothelial growth factor receptor 3 (VEGFR-3), Prospero homeobox protein 1 (Prox1), lymphatic vessel endothelial hyaluronan receptor 1 (LYVE-1), and podoplanin. In addition to maintaining fluid homeostasis and transporting lipids, lymphatic vessels (LVs) also regulate immune cell trafficking, antigen presentation, and immune monitoring 1, 2. Lymphatic vessels in adult mammals have been shown to be highly dynamic structures that interact closely with their microenvironment 1. Lymphangiogenesis involves the generation of new LVs from existing vessels, including the migration, proliferation, and luminal formation of LECs 3. The cellular mechanism of lymphangiogenesis, a characteristic feature of inflammatory diseases and tumors 4, is still unclear, and the evidence shows that both divisions of preexisting LECs and the incorporation of circulating lymphatic progenitors participate in the lymphangiogenic process 5. Our previous studies have shown that lymphangiogenesis in the kidney and its draining lymph nodes is related to preexisting C-C chemokine ligand 21 (CCL21)-producing LECs 6. Under normal conditions, LECs are in a static state. During tissue repair, inflammation, and tumor growth 7, 8, lymphatic endothelial cells migrate to related tissues in response to chemokine induction and proliferate to form lymphatic vessels with the help of growth factors, chemokines, growth factors, adhesion molecules, and the extracellular matrix 9. Macrophages 10-12, dendritic cells, and lymphocytes are known to coordinate lymphangiogenesis, and recent studies suggest that neutrophils also participate in this process 13, 14.

Emerging evidence suggests that neutrophils participate in regulating lymphangiogenesis by secreting lymphotropic factors or increasing their bioavailability 15-18. The vascular endothelial growth factor (VEGF) family and their ligands, VEGF receptors, are considered the most important molecules involved in the growth of new lymphatic vessels by lymphangiogenesis 19. The roles of VEGFA, VEGFC, and VEGFD in lymphangiogenesis are best understood 10, 11, 20-23. VEGFA induces lymphangiogenesis via VEGFR2, whereas VEGFC and VEGFD induce lymphangiogenesis through VEGFR2/3 signaling. VEGFC and VEGFD are crucial for the proliferation, migration, and survival of LECs 24. Mice inoculated intranasally with VEGFC and VEGFD exhibit the formation of lymphatic vessels, while inhibiting VEGFR-3 signaling prevents lymphatic vessel formation, with angiogenesis undisturbed 17. Neutrophils contribute to lymphangiogenesis by secreting VEGFC and VEGFD, increasing the biological activity of VEGFA and cooperating with other prolymphangiogenic cells 25. These cells surrounding the lymphatic vessels come into direct contact with the tumor and promote lymphatic angiogenesis 26.

Lymphangiogenesis plays a dual role in inflammation 27. Lymphangiogenic molecules not only directly affect inflammatory lymphangiogenesis but also contribute to chronic inflammation by recruiting more immune cells 28. Our previous studies also showed that lymphangiogenesis plays significant roles in mediating kidney inflammation and fibrosis 6. Several publications have explored potential therapies that target neutrophils directly or indirectly to address issues related to lymphangiogenesis. This review discusses the interaction between neutrophils and lymphangiogenesis in inflammation and tumors, the biological features of neutrophils in lymphangiogenesis, the mechanism by which neutrophils induce lymphangiogenesis, and neutrophil-related therapies.

## Biological features of neutrophils in lymphangiogenesis

### Recruitment of neutrophils

In addition to balancing fluid homeostasis and transporting fat, immune cell trafficking is considered one of the major functions of the lymphatic system. During immune activation, lymphatic vessels provide a route for antigen delivery and trafficking of T cells, dendritic cells, and neutrophils to draining lymph nodes (dLNs) 29. Accumulating evidence indicates that the interactions between lymphatic vessels and neutrophils may influence neutrophil migration, lymphatic vessel growth, and certain immune reactions. Interestingly, neutrophil migration may depend on the neutrophil phenotype 30.

#### Neutrophil entry into lymphatic vessels

Neutrophils capture bacteria and antigens from peripheral tissue and transport them to dLNs through lymphatic vessels, and infected neutrophils have been detected in the lumen of lymphatic vessels by confocal microscopy 31, 32. Interestingly, lymph-migrating neutrophils have an extended lifespan 33. As reported, neutrophils migrate via lymphatic vessels more rapidly than DCs and macrophages, arriving in the dLNs approximately 12-72 hours earlier 31, 34. Compared with other leukocytes, neutrophils use a more complex and efficient mechanism to enter lymphatic capillaries. Instead of migrating through the established cellular junctions of LECs, neutrophils induce the enzymatic degradation and retraction of junctions, creating a large passage for migration 34. β2 integrins are involved in the process by which neutrophils migrate into afferent lymphatics from inflamed tissues 16. Initially, inflammatory chemokines such as CXCL8 (CXCL1 in mice) induce neutrophil chemotaxis toward and swarm over inflamed LECs, forming initial adhesive interactions with E-selectin, which are upregulated instantly, followed by more stable β2 integrin-mediated binding via ICAM-1 and VCAM-1 (expressed on LECs) 31. This process triggers the secretion of matrix metalloproteinases (MMPs) and exocytosis of the chemorepellent 12(S)-HETE, promoting transient focal junctional retraction beneath the adherent neutrophil swarm 35, and the resulting gaps in the endothelium greatly accelerate neutrophil migration. Moreover, the gaps are transient, exist, and resolve spontaneously; thus, they serve as portals for successive neutrophil migration. Neutrophil migration in lymphatic vessels also requires the complement receptor CD11b, which binds to β2 integrins, and local blockade or complete removal (using CD11b-deficient mice) inhibits this process 30. Whether CD11b modulation cooperates with ICAM-1 or other molecules remains to be studied.

Previous studies have indicated that CCR7 may participate in the migration of neutrophils to LNs 36. Samantha *et al.* reported that TNF-α orchestrates neutrophil migration to dLNs (mostly via afferent lymphatics) in a CCR7-dependent manner 37. Moreover, this study showed that TNF-α controls the crawling of neutrophils along the afferent lymphatic lumen toward gradient concentrations of CCL21 and that this directional crawling may be mediated by ICAM-1 upregulation on LECs 37. To date, the function of CCR7 in neutrophil trafficking remains unclear; in a model of *S. aureus*-induced neutrophil migration, neutrophil egress from the skin to dLNs depended on CXCR4 and CD11b but not CCR7 30. These contradictory outcomes suggest that the CCR7 pathway is stimulus dependent and that the molecular axis used for migration may be influenced by the neutrophil phenotype or activation pathway 38.

#### Neutrophils are recruited from the circulation via HEVs

Postcapillary venules in the LN cortex are referred to as "high endothelial venules" (HEVs) because of their remarkable height of endothelium, and HEVs can be found in all secondary lymphoid organs except for the spleen 39. Neutrophils can enter LNs not only through lymphatic vessels but also via HEVs 40. A confocal microscopy image of inguinal LNs after *S. aureus* infection confirmed that neutrophils can be recruited via HEVs 41. The membrane surface of neutrophils can express L-selectin, which binds to glycoproteins on endothelial cells and promotes neutrophil tethering and rolling. The L-selectin-PNAd interaction together with the PSGL1-P-selectin interaction are two pathways that recruit neutrophils via HEVs 40, 42, and blocking PSLG-1 or L-selectin reduces neutrophil migration to LNs to some degree 30, 40, 43. In addition, two main β2 integrins, LFA-1 (CD11a) and Mac-1 (CD11b), reportedly cooperate in neutrophil migration from the blood and lymph nodes to the LNs 40. LFA-1 mediates neutrophil adhesion, whereas Mac-1 promotes adhesion and subsequent luminal crawling 44. The CXCR4/CXCL12 axis controls both neutrophil lymphatic and blood migration, and neutrophils significantly upregulate CXCR4 expression after antigen challenge for subsequent migration 40.

### Neutrophil markers

In the bone marrow, neutrophils can be divided into three subsets: proliferative neutrophil precursors, nonproliferating immature neutrophils and mature neutrophils. Immature neutrophils are capable of entering the bloodstream and migrating toward the site of injury with the same efficiency as mature neutrophils during inflammation 45. In addition to being classified according to their provenance, neutrophils can also be classified according to their surface markers, chemokine receptors, granule proteins and transcription factors. Given the intricacy of the classification process, we will concentrate more on neutrophil subpopulations associated with lymphangiogenesis.

In gastric cancer, the high level of infiltrating CD15^+^ TANs (tumor-associated neutrophils) is related to the systemic inflammatory response and lymph node (LN) metastasis. Investigators have proposed that CXCR2^+^ neutrophils may in fact be the source of the CD15^+^ TANs. TANs in primary gastric tumors may spread through lymphatic vessels and participate in cancer-related lymphangiogenesis 46. A large-scale analysis of human carcinoma samples revealed that CD66b^+^ TANs repeatedly colonized carcinoma-draining lymph nodes. An analysis of the primary tumors of oral squamous cell carcinomas revealed that CD66^+^ TANs colocalized with tumor cells in areas of lymphangiogenesis 47. An increasing number of studies suggest that TANs can be classified into antitumorigenic “N1” and protumorigenic “N2” phenotypes according to their different states of activation, cytokine repertoires, and roles in tumor immunity 48, 49. N1 TANs exhibit antitumor cytotoxicity and immunostimulatory profiles and are characterized by increased levels of proinflammatory chemokines, including TNFα, ROS, Fas, IL-12, CCL3, CXCL9, CXCL10, and ICAM-1, and the capacity to recruit and activate CD8^+^ T cells 50, 51. N2 TANs display an anti-inflammatory phenotype with the upregulation of chemokines such as CCL2, CCL5, and CXCL1, 2, 8 and 16 52. Compared with N1 TANs, N2 TANs express higher levels of MMP9, VEGFA, CXCR4, arginase, and CCL2, stimulating tumor angiogenesis. The presence of the N1 phenotype and N2 phenotype not only is limited to tumors but also occurs in inflammatory diseases. Among neutrophils isolated from patients with systemic lupus erythematosus, the number of N1 neutrophils is significantly increased, and N1 neutrophils secrete more TNF-α and IFN-γ, inhibiting endothelial progenitor cell differentiation and disrupting vascular repair 53.

## The roles of neutrophil-induced lymphangiogenesis in tumors and inflammation

Although the interaction between neutrophils and angiogenesis has been widely studied 54, 55, the role of neutrophils in lymphangiogenesis is only beginning to be revealed. I describe the regulatory mechanism of neutrophils on lymphangiogenesis from the aspects described below.

### Neutrophils are the source of VEGFC and VEGFD and contribute to lymphangiogenesis

Neutrophils have been reported to be a source of lymphangiogenic factors (VEGFC/D), which contribute to lymphangiogenesis in individuals with COPD 18. CircDHTKD1-induced CXCL5 expression was found to recruit and activate neutrophils, which are involved in lymphangiogenesis through the secretion of VEGFC, in studies of bladder cancer 56. In a model of* Mycoplasma pulmonis* infection in mice, VEGFD immunoreactivity was strongly detected in neutrophils, suggesting that neutrophils are cellular sources of VEGFD during inflammation 17. Neutrophils contribute to lymphangiogenesis by secreting VEGFD 13. Decreases in the total VEGFD levels and lymphatic vessel density (LVD),are diminished in the absence of neutrophils, whereas blocking VEGFD signaling can inhibit lymphangiogenesis at inflamed sites. Recent research has revealed that VEGFD not only promotes the proliferation of LECs but also modulates the morphology of collecting lymphatics 57.

Proteolytic processing is known as a regulator of VEGFC activity, and neutrophils may regulate the proteolytic processing of VEGFC and its bioavailability 58. Lu *et al.* reported that TANs recruited by CXCL5 contribute to lymphangiogenesis by secreting VEGFC 56. VEGFC-deficient embryos have shown the critical role of VEGFC in inducing the migration of LECs, as the failure of LECs to detach from the cardinal vein can cause embryonic lethality 59. VEGFC/VEGFR3 regulates the remodeling and homeostasis of lymphatic vessels and changes the contraction of lymphatic vessels via the surrounding smooth muscle 24, 60.

### Neutrophils increase the amount of biologically active VEGFA and contribute to lymphangiogenesis

Neutrophils contribute to lymphangiogenesis by increasing the amount of active VEGFA 13. Several isoforms of VEGFA are formed through differential mRNA splicing, and these isoforms differ in the heparin-binding domain (HBD), which mediates the interactions of VEGFA with the extracellular matrix (ECM), cell surface heparan sulfate proteoglycans, and neuropilin-1 61. Approximately 60-70% of VEGFA164, the most abundant and biologically active isoform, is anchored to the ECM, leaving a small portion of secreted VEGFA to remain biologically active 62. Angiogenesis-related studies have reported that neutrophils secrete MMP9 and heparinase, which cleave heparan sulfate proteoglycans (HSPs) side chains to release the HSP-trapped VEGFA 55, 63. The same mechanism may also apply to lymphangiogenesis, as the levels of the VEGFA-VEGFR2 complex, MMP9, and heparinase decrease in the absence of neutrophils 13. In summary, neutrophils secrete MMP9 and heparinase, release HSP-trapped VEGFA and increase VEGFA bioavailability, which contribute to lymphangiogenesis. VEGFA is also capable of stimulating lymphatic vessel expansion 64 and the development of high endothelial venules 65.

The CXCL1/8-CXCR2 and ERK/JNK pathways are relevant to neutrophil-derived VEGFA. Recent studies have shown that the infiltration of TANs increases the levels of VEGFA and MMP9 via the ERK/JNK pathway, facilitating lymphangiogenesis in bladder cancer 14. VEGFA plays an essential role in neutrophil-dependent lymphangiogenesis, and publications have reported that neutralizing VEGFA alone can cause the same extent of lymphangiogenesis observed with neutrophil depletion 13. TANs directly promote the formation of HLECs, while neutralizing VEGFA or inhibiting MMP activity can abolish the tube formation of HLECs 14.

### Neutrophil extracellular traps (NETs) and lymphangiogenesis

NETs are intricate network structures composed of DNA‒histone complexes and proteins released by neutrophils. While the effect of NETs on angiogenesis has been well studied, their connection with lymphangiogenesis is still poorly understood. Kawasaki disease is an acute systemic vasculitis syndrome. PBMCs from patients with Kawasaki disease expressed higher levels of VEGFA and HIF-1α than did PBMCs from healthy control patients after 24 hours of incubation with NETs from patients with Kawasaki disease. NETs may alter the biological response of PBMCs and lead to vascular damage in patients with Kawasaki disease; however, the specific mechanism is still unclear 66. In a recent study, the authors performed both *in vivo* and *in vitro* tests to show that NETs promote lymphangiogenesis and lymphatic permeability 67. Using PMA to stimulate primary human blood neutrophils to produce NETs and DNase1 to clear NETs, the researchers found that the number and length of neoplastic lymphatic vessels were significantly increased in the presence of NETs. Moreover, the researchers induced NET formation in BALB/c nude mice by injecting lipopolysaccharide (LPS). LYVE-1 staining of mouse lymph nodes revealed significantly increased lymphangiogenesis in LPS-treated mice. In hydrocephalus, NETs are involved in acute lymphatic endothelial cell injury and lymphatic vessel thrombosis 68.

### Neutrophil-induced lymphangiogenesis is associated with the tumor prognosis

Lymphangiogenesis is mostly discussed in cancer research because of its importance in cancer immunity. Lymphangiogenesis occurs at the margin areas of tumors or dLNs in several types of cancer, and those margin lymphatic vessels are capable of trafficking immune cells and draining tissue fluid, as well as mediating lymphatic metastasis 69. The mechanisms underlying lymphatic vessel remodeling and lymphangiogenesis are considered to promote lymph flow, helping tumor cells spread to sentinel LNs and distant organs 60. Lymphangiogenesis has been shown to be correlated with cancer metastasis and shorter disease-free survival 25. However, tumor cell metastasis through lymphangiogenesis may also result in tumor cells being exposed to the immune system. In addition, certain factors produced during lymphangiogenesis may inhibit the growth and survival of tumor cells 70. Abnormal expression of lymphangiogenic growth factors, such as VEGFC and VEGFD, can be useful early indicators for diagnosing tumor cell metastasis to LNs and distant organs 25. The parameters of lymphangiogenesis and lymphatic remodeling are related to the prognosis of patients with different cancers 71. Early research reported a strong correlation between a high VEGFD level and shorter survival of patients with endometrial carcinoma 72, 73. Meta-analyses of breast cancer 74, 75, non-small cell lung cancer 76-78, colorectal cancer 79, and esophageal carcinoma 80 have also reported that high levels of VEGFC/D and a high lymphatic vascular density are significant prognostic indicators of poor outcomes. Some researchers have reported no significant relationship, which could be explained by the complex roles of VEGFC/D in tumor immunity. Increasing evidence has revealed additional functions of VEGFC beyond the lymphatic system, such as regulating macrophages 81, NK cells 82, and platelet generation by megakaryocytes 83, which can affect tumor immunity in various ways. CXCL5 overexpression is significantly associated with melanoma LN metastasis in the clinic. Interestingly, in severe combined immunodeficient mice, CXCL5 recruits large numbers of neutrophils, which surround LVs and are often in direct contact with tumor cells, together with increased lymphangiogenesis 26. Neutrophils can respond to CXCL1 and CXCL8 secreted by tumor cells through the CXCR2 receptor, activating the ERK/JNK signaling pathway. This process promotes the secretion of VEGFA and MMP9 by neutrophils, regulating lymphangiogenesis and thereby facilitating the lymphatic metastasis of bladder cancer 84. A retrospective analysis was conducted on a cohort of 182 patients diagnosed with metastatic oral squamous cell carcinoma. The analysis revealed that a high CD66b^+^ TAN density in the metastatic tumor-draining lymph nodes was significantly associated with a worse prognosis 47.

### Neutrophil-induced lymphangiogenesis plays a dual role in inflammation

Lymphangiogenesis plays a dual role in inflammation. In the early stage of inflammation, lymphangiogenesis helps increase lymphatic fluid drainage and the trafficking of inflammatory mediators and immune cells, contributing to a decrease in inflammation 27. However, persistent lymphangiogenesis may contribute to chronic inflammation by transporting antigens or leukocytes, thus sustaining immune activation 27.

Human LECs express the CCR7 ligand CCL21 to regulate the transport of lymphocytes and DCs 85 and express receptors for proinflammatory β-chemokines, which contribute to leukocyte recirculation via the lymphatic system 86. The inhibition of lymphangiogenesis in mice infected with *M. pulmonis* predisposes them to bronchial lymphedema and exacerbated airflow obstruction 17. A similar conclusion was also reached for UVB-induced edema, suggesting a positive role of lymphangiogenesis in lymphedema 87. By using keratin 14-VEGFC transgenic mice to construct an acute skin inflammation model, researchers have shown that lymphangiogenesis helps promote antigen clearance and inflammation resolution 10. Lymphangiogenic molecules not only influence inflammatory lymphangiogenesis directly but also contribute to chronic inflammation by recruiting additional immune cells 88. VEGFA expression is upregulated in inflammatory diseases such as psoriasis 89, delayed-type hypersensitivity reactions, and rheumatoid arthritis. Experiments using VEGFA transgenic mice revealed the role of VEGFA in promoting persistent chronic inflammation by recruiting leukocytes, enlarging lymph, and improving vascular permeability 90. Corneal allografts are generally considered excellent areas for studying the effects of lymphangiogenesis because of the absence of blood and lymphatic vessels in healthy corneas. Inflammation following corneal allograft surgery induces lymphangiogenesis, disrupting the original "immune-privileged" status. Statistics show that the inhibition of lymphangiogenesis can improve graft survival in a murine corneal transplantation model through strategies such as neutralizing VEGFs and VEGFR 91-93. Studies related to renal transplant rejection suggest that lymphangiogenesis is associated with lymphocyte-rich inflammation caused by CCL21 expressed during renal transplantation, which elicits alloantigen recognition events 94. Inhibiting VEGFR3 can reduce CCL21 production and lymphocyte infiltration in cardiac allografts 95.

## Neutrophils cooperate with different immune cells to affect lymphangiogenesis

### Macrophages

Neutrophils cooperate with macrophages to promote lymphangiogenesis. The role of macrophages in lymphangiogenesis is well known. Our recent study also proved that macrophages interact with LECs by secreting CD137L to promote lymphangiogenesis in the kidney 96. These compelling data indicated that macrophages are indispensable in driving lymphangiogenesis in tissues and LNs via VEGFC, VEGFD, and VEGFA 10, 17, 97, and our previous research suggested that the VEGFC/VEGFR3 pathway promotes macrophage M1 polarization and transdifferentiation into LECs 98. Neutrophils are essential for activating and recruiting macrophages to inflamed sites, and the cycle continues with macrophages recruiting neutrophils 99. Rat corneal alkali injury is accompanied by neovascularization and lymphangiogenesis; early neutrophil infiltration peaks on the first day, whereas macrophage infiltration peaks on Day 7 100, suggesting a key role for neutrophils in the formation of lymphatic vessels. In an inflammatory lymphangiogenesis model in zebrafish, both macrophages and neutrophils express VEGFA, VEGFC, and VEGFD, contributing to lymphangiogenesis through the VEGFR pathway 101.

### Lymphocytes

Evidence for cross-talk between human neutrophils and lymphocytes during lymphangiogenesis has been reported 102. The effect of neutrophils on T cells is caused by a complex series of events, including influencing the level of antigen presentation, modulating APC maturation and function, and activating or inhibiting T-cell migration 30, 103, 104. Supernatants of activated human neutrophils recruit Th17 cells via the chemokines CCL2 and CCL20, whereas activated Th17 cells attract neutrophils by releasing CXCR8 105. Mature T cells drive lymphangiogenesis in the thyroid of mice overexpressing CCL21 106. However, T cells are also reported to negatively regulate the formation of LVs in LNs by secreting interferon-γ, which suppresses the expression of lymphatic-specific genes in LECs 107. These contrasting outcomes may be explained by the different subsets of T cells involved in secondary and tertiary lymphoid organ functions 107.

Neutrophils modulate the B-cell response directly by secreting cytokines and promoting B-cell survival and maturation 102. B cells promote lymphatic growth in LNs after immunization 108, 109 by expressing VEGFA 65. Neutrophils compensate for the ability of B cells to drive lymphangiogenesis in μMT transgenic mice that lack B cells 13.

### Mast cells and dendritic cells

Human mast cells are both sources and targets of lymphangiogenesis factors 28, 110, and express VEGFA, VEGFB, VEGFC, VEGFD, VEGFR1, and VEGFR2 88. Human lung mast cells constitutively express different VEGFA isoforms (121, 165, 189, and 206) and release them after IgE stimulation 110. Prostaglandin E2 and substance P can induce the production of VEGFA in human mast cells 111, 112. Mast cells drive lymphangiogenesis in gastric cancer through the release of VEGFC and VEGFF 113.

Conclusions remain to be drawn regarding the role of dendritic cells in lymphangiogenesis. A distinct disease model proved that dendritic cells express VEGFC/VEGFD and VEGFR-3 and upregulate their expression under inflammatory stimulation 17, 114. DCs induce VEGFR expression in the Cynops dorsal iris, which can accelerate lymphangiogenesis.

## Neutrophil-targeted therapy

Inhibiting the supply of neutrophils for delivery to lymphatic organs is an effective strategy for treating a series of inflammatory diseases 115, 116 and tumors 117. In a mouse model of pulmonary fibrosis induced by *Paracoccidioides brasiliensis* infection, the use of an anti-neutrophil monoclonal antibody could reduce fibrosis and the contents of IL-17, Th-17, and Treg cells 116, thus alleviating chronic inflammation. The depletion of neutrophils may have modulated ETV4 overexpression-induced lymphangiogenesis in footpad tumors. These findings suggest that ETV4 (ETS Variant Transcription Factor 4) might promote lymphangiogenesis in a TAN-dependent manner. However, depleting neutrophils in the ETV4-overexpressing group did not completely inhibit ETV4 overexpression-induced LN metastasis 84.

Given the irreplaceable role of neutrophil populations in the immune system, identifying and targeting neutrophil subtypes associated with lymphatic angiogenesis may be a better solution. The inhibition of neutrophil Bv8/PROK2 expression abrogates resistance to anti-VEGF antibodies in genetic models of colorectal cancer 118. Neutrophils are recruited to LNs in two ways, migrating through LVs or via high HEVs, and the two types of neutrophils have distinct phenotypes and functions. Neutrophils recruited through LVs exhibit an APC-like phenotype; they capture antigens and transport them to LNs 31, where they mediate rapid cellular communication and modulate the adaptive immune response 30, 119. Neutrophils transported via HEVs comprise a greater proportion of LN neutrophils, and this proportion of LN neutrophils composes an innate barrier 120, preventing the systemic dissemination of pathogens 42.

Identifying the specific neutrophil-derived factors involved in lymphangiogenesis may provide a more sufficient solution for neutrophil-targeted therapy. Some VEGF-VEGFR pathway inhibitors have been established and approved for clinical use as antiangiogenic agents by the FDA, but only modest efficacy has been observed in patients 18, 121. In the context of sepsis-induced cardiomyopathy, the administration of VEGFC-156S promotes lymphangiogenesis and neutrophil migration to the lymph nodes 122. Furthermore, side effects of VEGF inhibitors used in the clinic have been reported, including hemorrhage, atherosclerosis, proteinuria hypertension, and leuko-encephalopathy syndrome 123.

More pathways and targets need to be studied to explore better therapies that combine both effectiveness and safety. Some targets are associated with neutrophil infiltration and lymphangiogenesis. FoxC2 is highly expressed during the development and organization of lymphatic vessels, and the downregulation of FoxC2 leads to increased severity and susceptibility to experimental colitis, as well as increased neutrophil infiltration and lymphangiectasia 124. The opposite outcome was observed in Ang-2^-/-^ mice, in which neutrophil infiltration was reduced and inflammation-induced lymphangiogenesis was blocked 124. Cathepsin L (Ctsl) is a potential therapeutic target for controlling inflammatory responses in a wide range of disease states. Compared with Ctsl^+/+^ mice, Ctsl^-/-^ mice are more sensitive to pneumonia, as they are neutrophil rich, and less lymphangiogenesis occurs in the acute phase 125.

## Conclusions

In summary, increasing evidence supports the close relationship between lymphatic vessels and neutrophils. Neutrophils can enter LNs through two distinct pathways, and those involved in lymphangiogenesis may exhibit unique phenotypes. Neutrophils not only serve as a source of VEGFs but also increase the level of biologically active VEGFA. By interacting with other lymphangiogenic cells, neutrophils can directly or indirectly promote lymphangiogenesis under pathological conditions. However, many details remain to be elucidated, as neutrophil-derived cytokines are both predictors and therapeutic targets of lymphangiogenesis in inflammation and tumors. We believe that further analysis of the interactions between neutrophils and lymphangiogenesis could provide new insights into potential treatment strategies for related diseases.

## Figures and Tables

**Figure 1:**
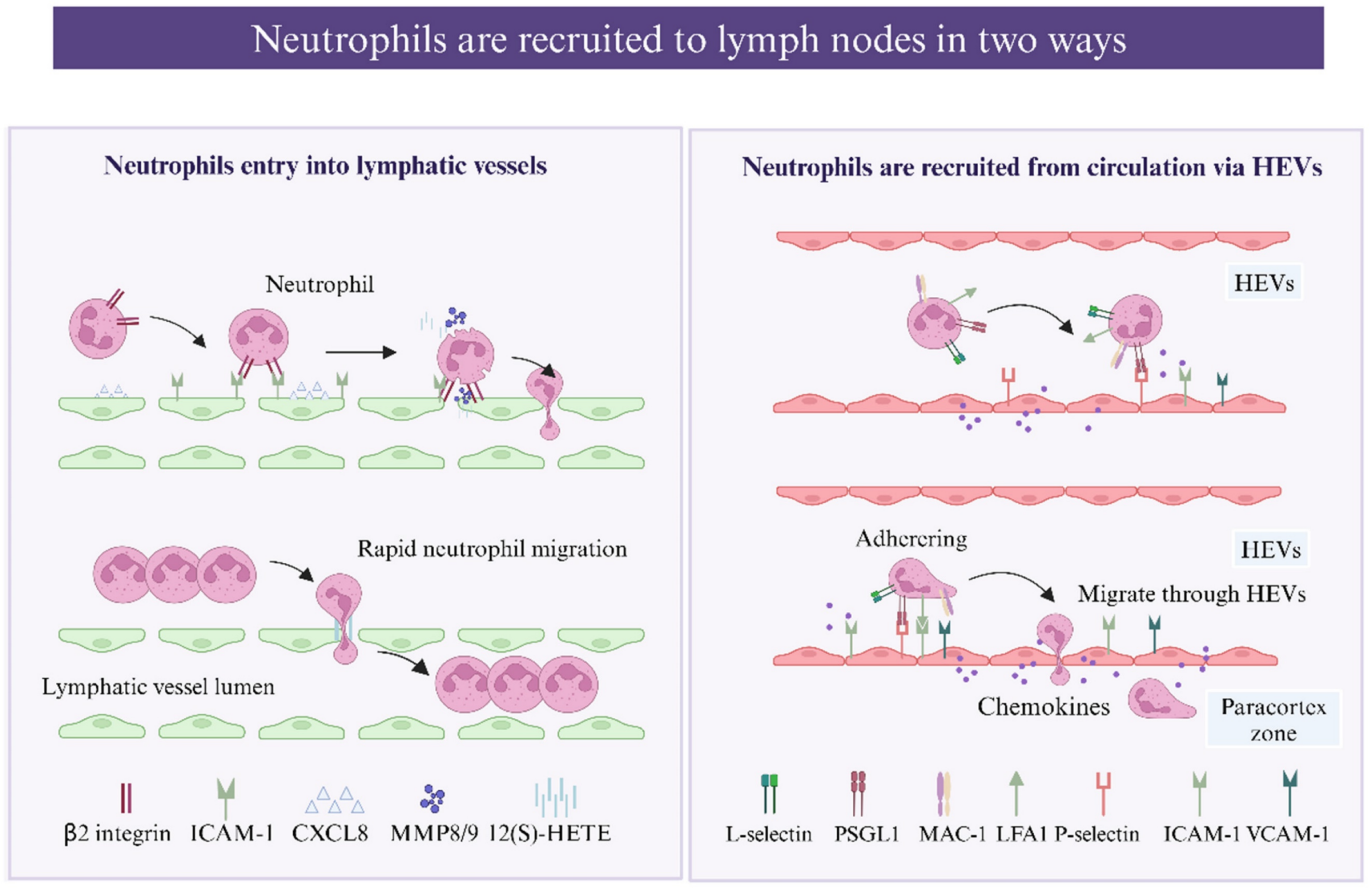
** Schematic representation of neutrophils be recruited to the lymph nodes** (created with BioRender.com). Neutrophils migrate to lymph nodes through the lymphatic vessels. Neutrophils chemotaxis toward inflamed LECs following the induction of CXCL8(CXCL1 in mice), forming adhesive interactions with LECs via β2 integrin-mediated binding via ICAM-1 and VCAM-1 (expressed on LECs). This process triggers the secretion of matrix metalloproteinases (MMPs) and exocytosis of the chemorepellent 12(S)-HETE. MMPs together with 12(S)-HETE induce the transient retraction of LEC-LEC junctions, and the resulting gaps in the endothelium greatly accelerate neutrophil migration. Neutrophils can enter LNs not only through lymphatic vessels but also via HEVs. L-selectin-PNAd interaction and PSGL-1-P-selectin interaction promote neutrophil tethering and rolling on venules. Neutrophil adhesion depends on a series of chemokines, such as IL-8, which bind to G-protein-coupled receptors (GPCRs), promoting the binding of ICAM-1 and VCAM-1 expressed on endothelial cells to integrins on the neutrophil surface. Two main β2 integrins, LFA-1 (CD11a) and Mac-1 (CD11b) are involved: LFA-1 mediates neutrophil adhesion, while Mac-1 promotes adhesion and subsequent luminal crawling.

**Figure 2:**
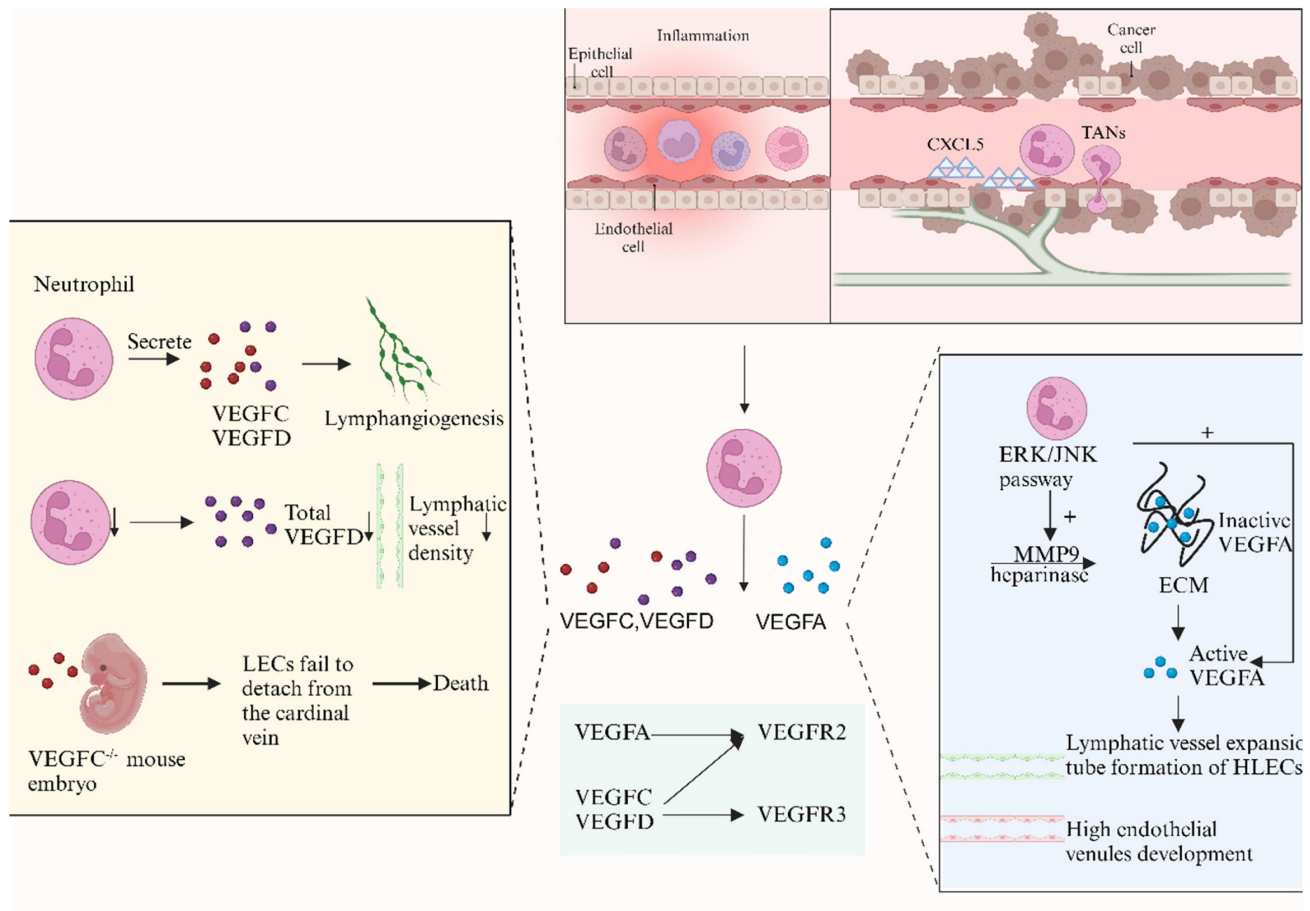
** Neutrophils contribute to lymphangiogenesis through the VEGF-VEGFR pathway** (created with BioRender.com). The VEGF-VEGFR pathway is considered the most important molecular mechanism in lymphangiogenesis. Studies show that neutrophils are cellular sources of VEGFD in inflammation and may regulate the proteolytic processing and bioavailability of VEGFC. Lu et al. reported that tumor-associated neutrophils (TANs) recruited by CXCL5 contribute to lymphangiogenesis by secreting VEGFC. When neutrophil levels decrease, total VEGFD and lymphatic vessel density (LVD) are reduced. Neutrophils also secrete MMP9 and heparinase, releasing HSP-trapped VEGFA and enhancing its bioavailability, thereby promoting lymphangiogenesis. Recent studies have shown that TANs infiltration increases VEGFA and MMP9 levels via the ERK/JNK pathway, facilitating lymphangiogenesis in bladder cancer. TANs directly affect human lymphatic endothelial cell (HLEC) formation, while neutralizing VEGFA or inhibiting MMP activity can eliminate HLEC tubular formation.

**Figure 3:**
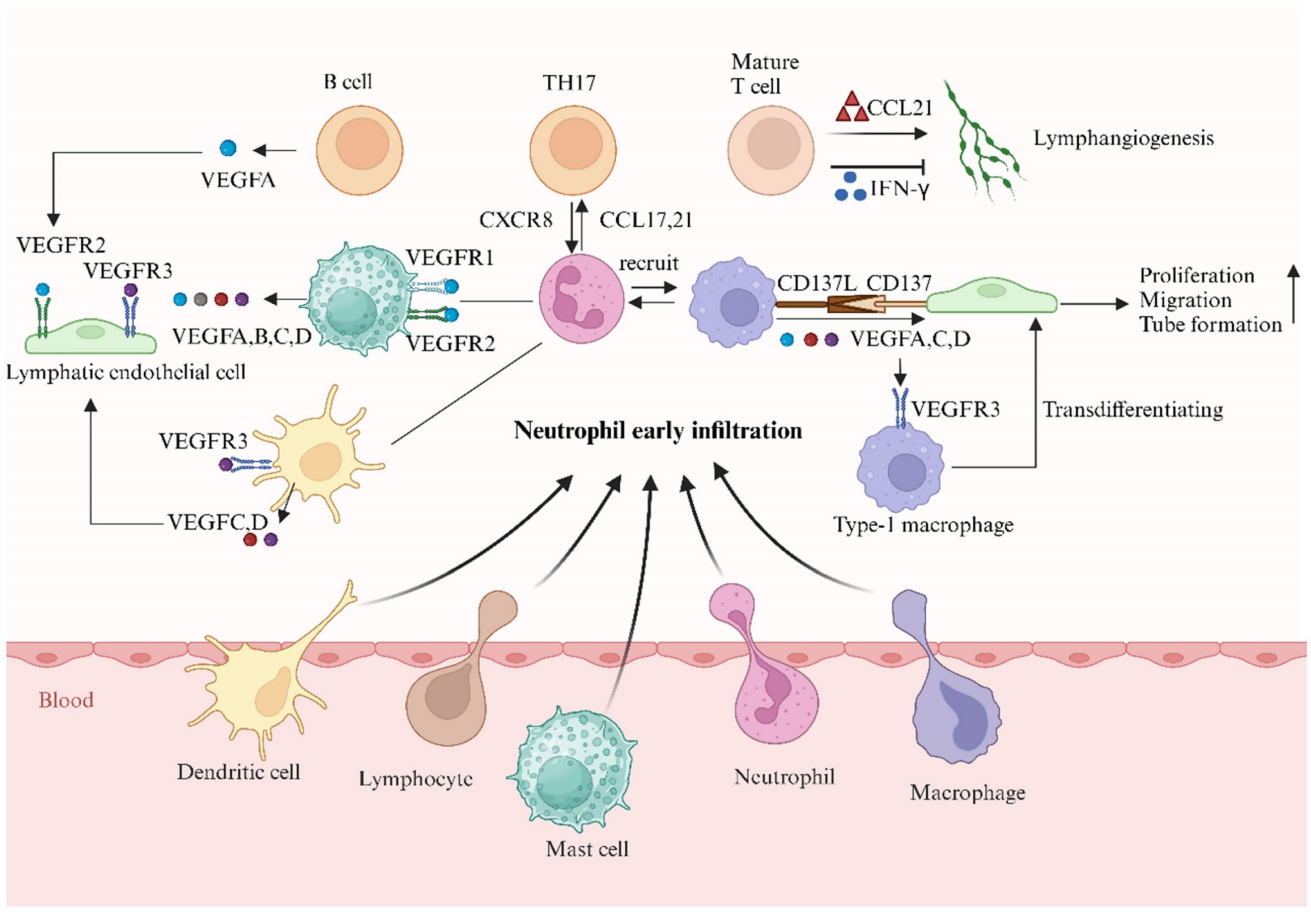
** Neutrophils collaborate with different immune cells to affect lymphangiogenesis** (created with BioRender.com). Neutrophils are essential in activating and recruiting immune cells to inflamed sites. In Rat cornea alkali injury, early neutrophil infiltration peaked on the first day while macrophage infiltration peaked on day 7, highlighting the key role of neutrophils in lymphatic vessel formation. The role of macrophages in lymphangiogenesis is well recognized. Our study also found that macrophages promote lymphangiogenesis in the kidney by secreting CD137L. Compelling data demonstrated macrophages are indispensable in driving lymphangiogenesis in tissues and LNs via VEGFC, VEGFD, and VEGFA. Meanwhile, the VEGFC-VEGFR3 pathway promotes macrophage M1 polarization and transdifferentiation into LECs. Mast cells and dendritic cells are both sources and targets of lymphangiogenesis factors. Evidence suggests there is a cross-talk between human neutrophils and lymphocytes during lymphangiogenesis. Activated human neutrophils recruit Th17 cells via CCL2 and CCL20 chemokines, while Th17 cells attract neutrophils by releasing CXCL8. T cells exhibit both positive and negative effects on lymphangiogenesis. Mature T cells drive lymphangiogenesis in the thyroid of mice overexpressing CCL21, but they also negatively regulate lymphatic vessel (LV) formation in LNs by secreting interferon-γ, which suppresses the expression of lymphatic-specific genes in LECs.

## Abbreviations

CCL21: C-C chemokine ligand 21
Ctsl: Cathepsin L
dLNs: Draining lymph nodes
ECM: Extracellular matrix
ETV4: ETS Variant Transcription Factor 4
HBD: Heparin-binding domain
HSP: Heparan sulfate proteoglycans
LECs: Lymphatic endothelial cells
LNs: Lymph nodes
LPS: Lipopolysaccharide
LYVE-1: Lymphatic vessel endothelial hyaluronan receptor 1
LVs: Lymphatic vessels
MMP: Matrix metalloproteinase
NETs: Neutrophil extracellular traps
Prox1: Prospero homeobox protein 1
TANs: Tumor-associated neutrophils
VEGF: Vascular endothelial growth factor
VEGFR: Vascular endothelial growth factor receptor

## References

[1] Alitalo K (2011). The lymphatic vasculature in disease. Nature medicine.

[2] Pan X, Li X, Dong L, Liu T, Zhang M, Zhang L (2024). Tumour vasculature at single-cell resolution. Nature.

[3] Tan KW, Chong SZ, Angeli V (2014). Inflammatory lymphangiogenesis: cellular mediators and functional implications. Angiogenesis.

[4] Dieterich LC, Seidel CD, Detmar M (2014). Lymphatic vessels: new targets for the treatment of inflammatory diseases. Angiogenesis.

[5] Kerjaschki D, Huttary N, Raab I, Regele H, Bojarski-Nagy K, Bartel G (2006). Lymphatic endothelial progenitor cells contribute to *de novo* lymphangiogenesis in human renal transplants. Nature medicine.

[6] Pei G, Yao Y, Yang Q, Wang M, Wang Y, Wu J (2019). Lymphangiogenesis in kidney and lymph node mediates renal inflammation and fibrosis. Science advances.

[7] Vigl B, Aebischer D, Nitschké M, Iolyeva M, Röthlin T, Antsiferova O (2011). Tissue inflammation modulates gene expression of lymphatic endothelial cells and dendritic cell migration in a stimulus-dependent manner. Blood.

[8] Tammela T, Alitalo K (2010). Lymphangiogenesis: Molecular mechanisms and future promise. Cell.

[9] Kim H, Kataru RP, Koh GY (2012). Regulation and implications of inflammatory lymphangiogenesis. Trends in immunology.

[10] Kataru RP, Jung K, Jang C, Yang H, Schwendener RA, Baik JE (2009). Critical role of CD11b+ macrophages and VEGF in inflammatory lymphangiogenesis, antigen clearance, and inflammation resolution. Blood.

[11] Cursiefen C, Maruyama K, Bock F, Saban D, Sadrai Z, Lawler J (2011). Thrombospondin 1 inhibits inflammatory lymphangiogenesis by CD36 ligation on monocytes. The Journal of experimental medicine.

[12] Kang S, Lee SP, Kim KE, Kim HZ, Mémet S, Koh GY (2009). Toll-like receptor 4 in lymphatic endothelial cells contributes to LPS-induced lymphangiogenesis by chemotactic recruitment of macrophages. Blood.

[13] Tan KW, Chong SZ, Wong FH, Evrard M, Tan SM, Keeble J (2013). Neutrophils contribute to inflammatory lymphangiogenesis by increasing VEGF-A bioavailability and secreting VEGF-D. Blood.

[14] Luo R, Cheng Y, Chang D, Liu T, Liu L, Pei G (2021). Tertiary lymphoid organs are associated with the progression of kidney damage and regulated by interleukin-17A. Theranostics.

[15] Veikkola T, Jussila L, Makinen T, Karpanen T, Jeltsch M, Petrova TV (2001). Signalling via vascular endothelial growth factor receptor-3 is sufficient for lymphangiogenesis in transgenic mice. The EMBO journal.

[16] Rigby DA, Ferguson DJ, Johnson LA, Jackson DG (2015). Neutrophils rapidly transit inflamed lymphatic vessel endothelium via integrin-dependent proteolysis and lipoxin-induced junctional retraction. Journal of leukocyte biology.

[17] Baluk P, Tammela T, Ator E, Lyubynska N, Achen MG, Hicklin DJ (2005). Pathogenesis of persistent lymphatic vessel hyperplasia in chronic airway inflammation. The Journal of clinical investigation.

[18] Poto R, Loffredo S, Palestra F, Marone G, Patella V, Varricchi G (2022). Angiogenesis, Lymphangiogenesis, and Inflammation in Chronic Obstructive Pulmonary Disease (COPD): Few Certainties and Many Outstanding Questions. Cells.

[19] Lohela M, Bry M, Tammela T, Alitalo K (2009). VEGFs and receptors involved in angiogenesis versus lymphangiogenesis. Current Opinion in Cell Biology.

[20] Huggenberger R, Ullmann S, Proulx ST, Pytowski B, Alitalo K, Detmar M (2010). Stimulation of lymphangiogenesis via VEGFR-3 inhibits chronic skin inflammation. The Journal of experimental medicine.

[21] Huggenberger R, Siddiqui SS, Brander D, Ullmann S, Zimmermann K, Antsiferova M (2011). An important role of lymphatic vessel activation in limiting acute inflammation. Blood.

[22] Halin C, Tobler NE, Vigl B, Brown LF, Detmar M (2007). VEGF-A produced by chronically inflamed tissue induces lymphangiogenesis in draining lymph nodes. Blood.

[23] Kubo H, Cao R, Brakenhielm E, Mäkinen T, Cao Y, Alitalo K (2002). Blockade of vascular endothelial growth factor receptor-3 signaling inhibits fibroblast growth factor-2-induced lymphangiogenesis in mouse cornea. Proceedings of the National Academy of Sciences of the United States of America.

[24] Karaman S, Leppänen VM, Alitalo K (2018). Vascular endothelial growth factor signaling in development and disease. Development (Cambridge, England).

[25] Stacker SA, Williams SP, Karnezis T, Shayan R, Fox SB, Achen MG (2014). Lymphangiogenesis and lymphatic vessel remodelling in cancer. Nature reviews Cancer.

[26] Soler-Cardona A, Forsthuber A, Lipp K, Ebersberger S, Heinz M, Schossleitner K (2018). CXCL5 Facilitates Melanoma Cell-Neutrophil Interaction and Lymph Node Metastasis. The Journal of investigative dermatology.

[27] Aloisi F, Pujol-Borrell R (2006). Lymphoid neogenesis in chronic inflammatory diseases. Nature reviews Immunology.

[28] Varricchi G, Loffredo S, Galdiero MR, Marone G, Cristinziano L, Granata F (2018). Innate effector cells in angiogenesis and lymphangiogenesis. Current opinion in immunology.

[29] Randolph GJ, Ivanov S, Zinselmeyer BH, Scallan JP (2017). The Lymphatic System: Integral Roles in Immunity. Annual review of immunology.

[30] Hampton HR, Bailey J, Tomura M, Brink R, Chtanova T (2015). Microbe-dependent lymphatic migration of neutrophils modulates lymphocyte proliferation in lymph nodes. Nature communications.

[31] Abadie V, Badell E, Douillard P, Ensergueix D, Leenen PJ, Tanguy M (2005). Neutrophils rapidly migrate via lymphatics after Mycobacterium bovis BCG intradermal vaccination and shuttle live bacilli to the draining lymph nodes. Blood.

[32] Maletto BA, Ropolo AS, Alignani DO, Liscovsky MV, Ranocchia RP, Moron VG (2006). Presence of neutrophil-bearing antigen in lymphoid organs of immune mice. Blood.

[33] Kim MH, Granick JL, Kwok C, Walker NJ, Borjesson DL, Curry FR (2011). Neutrophil survival and c-kit(+)-progenitor proliferation in Staphylococcus aureus-infected skin wounds promote resolution. Blood.

[34] Teijeira A, Halin C (2015). Editorial: Breaching their way through: Neutrophils destroy intercellular junctions to transmigrate rapidly across lymphatic endothelium. Journal of leukocyte biology.

[35] Jackson DG (2019). Leucocyte Trafficking via the Lymphatic Vasculature- Mechanisms and Consequences. Frontiers in immunology.

[36] Beauvillain C, Cunin P, Doni A, Scotet M, Jaillon S, Loiry ML (2011). CCR7 is involved in the migration of neutrophils to lymph nodes. Blood.

[37] Arokiasamy S, Zakian C, Dilliway J, Wang W, Nourshargh S, Voisin MB (2017). Endogenous TNFα orchestrates the trafficking of neutrophils into and within lymphatic vessels during acute inflammation. Scientific reports.

[38] Jakovija A, Chtanova T (2021). Neutrophil Interactions with the Lymphatic System. Cells.

[39] Rosen SD (2004). Ligands for L-selectin: homing, inflammation, and beyond. Annual review of immunology.

[40] Gorlino CV, Ranocchia RP, Harman MF, García IA, Crespo MI, Morón G (2014). Neutrophils exhibit differential requirements for homing molecules in their lymphatic and blood trafficking into draining lymph nodes. Journal of immunology (Baltimore, Md: 1950).

[41] Kamenyeva O, Boularan C, Kabat J, Cheung GY, Cicala C, Yeh AJ (2015). Neutrophil recruitment to lymph nodes limits local humoral response to Staphylococcus aureus. PLoS pathogens.

[42] Bogoslowski A, Butcher EC, Kubes P (2018). Neutrophils recruited through high endothelial venules of the lymph nodes via PNAd intercept disseminating Staphylococcus aureus. Proceedings of the National Academy of Sciences of the United States of America.

[43] Brackett CM, Muhitch JB, Evans SS, Gollnick SO (2013). IL-17 promotes neutrophil entry into tumor-draining lymph nodes following induction of sterile inflammation. Journal of immunology (Baltimore, Md: 1950).

[44] Phillipson M, Heit B, Colarusso P, Liu L, Ballantyne CM, Kubes P (2006). Intraluminal crawling of neutrophils to emigration sites: a molecularly distinct process from adhesion in the recruitment cascade. The Journal of experimental medicine.

[45] Evrard M, Kwok IWH, Chong SZ, Teng KWW, Becht E, Chen J (2018). Developmental Analysis of Bone Marrow Neutrophils Reveals Populations Specialized in Expansion, Trafficking, and Effector Functions. Immunity.

[46] Hiramatsu S, Tanaka H, Nishimura J, Sakimura C, Tamura T, Toyokawa T (2018). Neutrophils in primary gastric tumors are correlated with neutrophil infiltration in tumor-draining lymph nodes and the systemic inflammatory response. BMC immunology.

[47] Lonardi S, Missale F, Calza S, Bugatti M, Vescovi R, Debora B (2021). Tumor-associated neutrophils (TANs) in human carcinoma-draining lymph nodes: a novel TAN compartment. Clinical & translational immunology.

[48] Fridlender ZG, Sun J, Kim S, Kapoor V, Cheng GJ, Ling LN (2009). Polarization of Tumor-Associated Neutrophil Phenotype by TGF-beta: "N1" versus "N2" TAN. Cancer Cell.

[49] Houghton AM (2010). The paradox of tumor-associated neutrophils Fueling tumor growth with cytotoxic substances. Cell Cycle.

[50] Ohms M, Moller S, Laskay T (2020). An Attempt to Polarize Human Neutrophils Toward N1 and N2 Phenotypes *in vitro*. Frontiers in immunology.

[51] Sionov RV, Fridlender ZG, Granot Z (2015). The Multifaceted Roles Neutrophils Play in the Tumor Microenvironment. Cancer microenvironment: official journal of the International Cancer Microenvironment Society.

[52] Shirasuna K, Shimizu T, Matsui M, Miyamoto A (2013). Emerging roles of immune cells in luteal angiogenesis. Reproduction Fertility and Development.

[53] Denny MR, Yalavarthi S, Zhao WP, Thacker SG, Anderson M, Sandy AR (2010). A Distinct Subset of Proinflammatory Neutrophils Isolated from Patients with Systemic Lupus Erythematosus Induces Vascular Damage and Synthesizes Type I IFNs. Journal of Immunology.

[54] Ribatti D, Crivellato E (2009). Immune cells and angiogenesis. Journal of cellular and molecular medicine.

[55] Poto R, Cristinziano L, Modestino L, de Paulis A, Marone G, Loffredo S (2022). Neutrophil Extracellular Traps, Angiogenesis and Cancer. Biomedicines.

[56] Lu Q, Yin H, Deng Y, Chen W, Diao W, Ding M (2022). circDHTKD1 promotes lymphatic metastasis of bladder cancer by upregulating CXCL5. Cell death discovery.

[57] Karnezis T, Shayan R, Caesar C, Roufail S, Harris NC, Ardipradja K (2012). VEGF-D promotes tumor metastasis by regulating prostaglandins produced by the collecting lymphatic endothelium. Cancer Cell.

[58] Joukov V, Sorsa T, Kumar V, Jeltsch M, Claesson-Welsh L, Cao Y (1997). Proteolytic processing regulates receptor specificity and activity of VEGF-C. The EMBO journal.

[59] Hägerling R, Pollmann C, Andreas M, Schmidt C, Nurmi H, Adams RH (2013). A novel multistep mechanism for initial lymphangiogenesis in mouse embryos based on ultramicroscopy. The EMBO journal.

[60] Gogineni A, Caunt M, Crow A, Lee CV, Fuh G, van Bruggen N (2013). Inhibition of VEGF-C modulates distal lymphatic remodeling and secondary metastasis. PloS one.

[61] Künnapuu J, Bokharaie H, Jeltsch M (2021). Proteolytic Cleavages in the VEGF Family: Generating Diversity among Angiogenic VEGFs, Essential for the Activation of Lymphangiogenic VEGFs. Biology.

[62] Markovic-Mueller S, Stuttfeld E, Asthana M, Weinert T, Bliven S, Goldie KN (2017). Structure of the Full-length VEGFR-1 Extracellular Domain in Complex with VEGF-A. Structure (London, England: 1993).

[63] Nozawa H, Chiu C, Hanahan D (2006). Infiltrating neutrophils mediate the initial angiogenic switch in a mouse model of multistage carcinogenesis. Proceedings of the National Academy of Sciences of the United States of America.

[64] Wirzenius M, Tammela T, Uutela M, He Y, Odorisio T, Zambruno G (2007). Distinct vascular endothelial growth factor signals for lymphatic vessel enlargement and sprouting. The Journal of experimental medicine.

[65] Shrestha B, Hashiguchi T, Ito T, Miura N, Takenouchi K, Oyama Y (2010). B cell-derived vascular endothelial growth factor A promotes lymphangiogenesis and high endothelial venule expansion in lymph nodes. Journal of immunology (Baltimore, Md: 1950).

[66] Jing Y, Ding M, Fu J, Xiao Y, Chen X, Zhang Q (2020). Neutrophil extracellular trap from Kawasaki disease alter the biologic responses of PBMC. Bioscience reports.

[67] Ning Y, Chen Y, Tian T, Gao X, Liu X, Wang J (2024). S100A7 orchestrates neutrophil chemotaxis and drives neutrophil extracellular traps (NETs) formation to facilitate lymph node metastasis in cervical cancer patients. Cancer letters.

[68] Zhang Q, Chen Y, Li Y, Feng Z, Liang L, Hao X (2024). Neutrophil extracellular trap-mediated impairment of meningeal lymphatic drainage exacerbates secondary hydrocephalus after intraventricular hemorrhage. Theranostics.

[69] Vaahtomeri K, Alitalo K (2020). Lymphatic Vessels in Tumor Dissemination versus Immunotherapy. Cancer research.

[70] Karakousi T, Mudianto T, Lund AW (2024). Lymphatic vessels in the age of cancer immunotherapy. Nature reviews Cancer.

[71] Doeden K, Ma Z, Narasimhan B, Swetter SM, Detmar M, Dadras SS (2009). Lymphatic invasion in cutaneous melanoma is associated with sentinel lymph node metastasis. Journal of cutaneous pathology.

[72] Yokoyama Y, Charnock-Jones DS, Licence D, Yanaihara A, Hastings JM, Holland CM (2003). Expression of vascular endothelial growth factor (VEGF)-D and its receptor, VEGF receptor 3, as a prognostic factor in endometrial carcinoma. Clinical cancer research: an official journal of the American Association for Cancer Research.

[73] Mondal DK, Xie C, Pascal GJ, Buraschi S, Iozzo RV (2024). Decorin suppresses tumor lymphangiogenesis: A mechanism to curtail cancer progression. Proceedings of the National Academy of Sciences of the United States of America.

[74] Wang J, Guo Y, Wang B, Bi J, Li K, Liang X (2012). Lymphatic microvessel density and vascular endothelial growth factor-C and -D as prognostic factors in breast cancer: a systematic review and meta-analysis of the literature. Molecular biology reports.

[75] Su F, Liu B, Chen M, Xiao J, Li X, Lv X (2016). Association between VEGF-A, C and D expression and lymph node involvement in breast cancer: a meta-analysis. The International journal of biological markers.

[76] Kilvaer TK, Paulsen EE, Hald SM, Wilsgaard T, Bremnes RM, Busund LT (2015). Lymphangiogenic Markers and Their Impact on Nodal Metastasis and Survival in Non-Small Cell Lung Cancer-A Structured Review with Meta-Analysis. PloS one.

[77] Wei D, Xin Y, Rong Y, Hao Y (2022). Correlation between the Expression of VEGF and Ki67 and Lymph Node Metastasis in Non-small-Cell Lung Cancer: A Systematic Review and Meta-Analysis. Evidence-based complementary and alternative medicine: eCAM.

[78] Deluce J, Maj D, Boldt G, Breadner D, Raphael J (2020). Efficacy and toxicity of combined inhibition of EGFR and VEGFR in advanced non-small cell lung cancer patients harboring activating EGFR mutations: A systematic review and meta-analysis. Annals of Oncology.

[79] Zong S, Li H, Shi Q, Liu S, Li W, Hou F (2016). Prognostic significance of VEGF-C immunohistochemical expression in colorectal cancer: A meta-analysis. Clinica chimica acta; international journal of clinical chemistry.

[80] Peng J, Shao N, Peng H, Chen LQ (2013). Prognostic significance of vascular endothelial growth factor expression in esophageal carcinoma: a meta-analysis. Journal of BUON: official journal of the Balkan Union of Oncology.

[81] Tacconi C, Ungaro F, Correale C, Arena V, Massimino L, Detmar M (2019). Activation of the VEGFC/VEGFR3 Pathway Induces Tumor Immune Escape in Colorectal Cancer. Cancer research.

[82] Lee JY, Park S, Min WS, Kim HJ (2014). Restoration of natural killer cell cytotoxicity by VEGFR-3 inhibition in myelogenous leukemia. Cancer letters.

[83] Thiele W, Krishnan J, Rothley M, Weih D, Plaumann D, Kuch V (2012). VEGFR-3 is expressed on megakaryocyte precursors in the murine bone marrow and plays a regulatory role in megakaryopoiesis. Blood.

[84] Zhang Q, Liu S, Wang H, Xiao K, Lu J, Chen S (2023). ETV4 Mediated Tumor-Associated Neutrophil Infiltration Facilitates Lymphangiogenesis and Lymphatic Metastasis of Bladder Cancer. Advanced science (Weinheim, Baden-Wurttemberg, Germany).

[85] Ohl L, Mohaupt M, Czeloth N, Hintzen G, Kiafard Z, Zwirner J (2004). CCR7 governs skin dendritic cell migration under inflammatory and steady-state conditions. Immunity.

[86] Collado-Diaz V, Medina-Sanchez JD, Gkountidi AO, Halin C (2022). Imaging leukocyte migration through afferent lymphatics. Immunological reviews.

[87] Kajiya K, Detmar M (2006). An important role of lymphatic vessels in the control of UVB-induced edema formation and inflammation. The Journal of investigative dermatology.

[88] Marone G, Varricchi G, Loffredo S, Granata F (2016). Mast cells and basophils in inflammatory and tumor angiogenesis and lymphangiogenesis. European journal of pharmacology.

[89] Luengas-Martinez A, Paus R, Young HS (2022). Antivascular endothelial growth factor-A therapy: a novel personalized treatment approach for psoriasis. The British journal of dermatology.

[90] Kunstfeld R, Hirakawa S, Hong YK, Schacht V, Lange-Asschenfeldt B, Velasco P (2004). Induction of cutaneous delayed-type hypersensitivity reactions in VEGF-A transgenic mice results in chronic skin inflammation associated with persistent lymphatic hyperplasia. Blood.

[91] Cursiefen C, Cao J, Chen L, Liu Y, Maruyama K, Jackson D (2004). Inhibition of hemangiogenesis and lymphangiogenesis after normal-risk corneal transplantation by neutralizing VEGF promotes graft survival. Investigative ophthalmology & visual science.

[92] Dietrich T, Bock F, Yuen D, Hos D, Bachmann BO, Zahn G (2010). Cutting edge: lymphatic vessels, not blood vessels, primarily mediate immune rejections after transplantation. Journal of immunology (Baltimore, Md: 1950).

[93] Bachmann BO, Luetjen-Drecoll E, Bock F, Wiegand SJ, Hos D, Dana R (2009). Transient postoperative vascular endothelial growth factor (VEGF)-neutralisation improves graft survival in corneas with partly regressed inflammatory neovascularisation. The British journal of ophthalmology.

[94] Kerjaschki D, Regele HM, Moosberger I, Nagy-Bojarski K, Watschinger B, Soleiman A (2004). Lymphatic neoangiogenesis in human kidney transplants is associated with immunologically active lymphocytic infiltrates. Journal of the American Society of Nephrology: JASN.

[95] Nykänen AI, Sandelin H, Krebs R, Keränen MA, Tuuminen R, Kärpänen T (2010). Targeting lymphatic vessel activation and CCL21 production by vascular endothelial growth factor receptor-3 inhibition has novel immunomodulatory and antiarteriosclerotic effects in cardiac allografts. Circulation.

[96] Wei H, Chen L, Li Q, Liang X, Wang K, Zhang Y (2022). CD137L-macrophage induce lymphatic endothelial cells autophagy to promote lymphangiogenesis in renal fibrosis. International journal of biological sciences.

[97] Watari K, Nakao S, Fotovati A, Basaki Y, Hosoi F, Bereczky B (2008). Role of macrophages in inflammatory lymphangiogenesis: Enhanced production of vascular endothelial growth factor C and D through NF-κB activation. Biochemical and Biophysical Research Communications.

[98] Zhang Y, Zhang C, Li L, Liang X, Cheng P, Li Q (2021). Lymphangiogenesis in renal fibrosis arises from macrophages via VEGF-C/VEGFR3-dependent autophagy and polarization. Cell death & disease.

[99] Selders GS, Fetz AE, Radic MZ, Bowlin GL (2017). An overview of the role of neutrophils in innate immunity, inflammation and host-biomaterial integration. Regenerative biomaterials.

[100] Choi H, Phillips C, Oh JY, Stock EM, Kim DK, Won JK (2017). Comprehensive Modeling of Corneal Alkali Injury in the Rat Eye. Current eye research.

[101] Okuda KS, Misa JP, Oehlers SH, Hall CJ, Ellett F, Alasmari S (2015). A zebrafish model of inflammatory lymphangiogenesis. Biology open.

[102] Costa S, Bevilacqua D, Cassatella MA, Scapini P (2019). Recent advances on the crosstalk between neutrophils and B or T lymphocytes. Immunology.

[103] Yang CW, Strong BS, Miller MJ, Unanue ER (2010). Neutrophils influence the level of antigen presentation during the immune response to protein antigens in adjuvants. Journal of immunology (Baltimore, Md: 1950).

[104] Beauvillain C, Delneste Y, Scotet M, Peres A, Gascan H, Guermonprez P (2007). Neutrophils efficiently cross-prime naive T cells *in vivo*. Blood.

[105] Pelletier M, Maggi L, Micheletti A, Lazzeri E, Tamassia N, Costantini C (2010). Evidence for a cross-talk between human neutrophils and Th17 cells. Blood.

[106] Furtado GC, Marinkovic T, Martin AP, Garin A, Hoch B, Hubner W (2007). Lymphotoxin beta receptor signaling is required for inflammatory lymphangiogenesis in the thyroid. Proceedings of the National Academy of Sciences of the United States of America.

[107] Kataru RP, Kim H, Jang C, Choi DK, Koh BI, Kim M (2011). T lymphocytes negatively regulate lymph node lymphatic vessel formation. Immunity.

[108] Angeli V, Ginhoux F, Llodrà J, Quemeneur L, Frenette PS, Skobe M (2006). B cell-driven lymphangiogenesis in inflamed lymph nodes enhances dendritic cell mobilization. Immunity.

[109] Liao S, Ruddle NH (2006). Synchrony of high endothelial venules and lymphatic vessels revealed by immunization. Journal of immunology (Baltimore, Md: 1950).

[110] Detoraki A, Staiano RI, Granata F, Giannattasio G, Prevete N, de Paulis A (2009). Vascular endothelial growth factors synthesized by human lung mast cells exert angiogenic effects. The Journal of allergy and clinical immunology.

[111] Abdel-Majid RM, Marshall JS (2004). Prostaglandin E2 induces degranulation-independent production of vascular endothelial growth factor by human mast cells. Journal of immunology (Baltimore, Md: 1950).

[112] Theoharides TC, Zhang B, Kempuraj D, Tagen M, Vasiadi M, Angelidou A (2010). IL-33 augments substance P-induced VEGF secretion from human mast cells and is increased in psoriatic skin. Proceedings of the National Academy of Sciences of the United States of America.

[113] Sammarco G, Varricchi G, Ferraro V, Ammendola M, De Fazio M, Altomare DF (2019). Mast Cells, Angiogenesis and Lymphangiogenesis in Human Gastric Cancer. International journal of molecular sciences.

[114] Hamrah P, Chen L, Zhang Q, Dana MR (2003). Novel expression of vascular endothelial growth factor receptor (VEGFR)-3 and VEGF-C on corneal dendritic cells. The American journal of pathology.

[115] Soehnlein O, Libby P (2021). Targeting inflammation in atherosclerosis - from experimental insights to the clinic. Nature reviews Drug discovery.

[116] Puerta-Arias JD, Pino-Tamayo PA, Arango JC, González Á (2016). Depletion of Neutrophils Promotes the Resolution of Pulmonary Inflammation and Fibrosis in Mice Infected with Paracoccidioides brasiliensis. PloS one.

[117] Leslie J, Mackey JBG, Jamieson T, Ramon-Gil E, Drake TM, Fercoq F (2022). CXCR2 inhibition enables NASH-HCC immunotherapy. Gut.

[118] Itatani Y, Yamamoto T, Zhong C, Molinolo AA, Ruppel J, Hegde P (2020). Suppressing neutrophil-dependent angiogenesis abrogates resistance to anti-VEGF antibody in a genetic model of colorectal cancer. Proceedings of the National Academy of Sciences of the United States of America.

[119] Duffy D, Perrin H, Abadie V, Benhabiles N, Boissonnas A, Liard C (2012). Neutrophils transport antigen from the dermis to the bone marrow, initiating a source of memory CD8+ T cells. Immunity.

[120] Bogoslowski A, Kubes P (2018). Lymph Nodes: The Unrecognized Barrier against Pathogens. ACS infectious diseases.

[121] Xu W, Harris NR, Caron KM (2021). Lymphatic Vasculature: An Emerging Therapeutic Target and Drug Delivery Route. Annual review of medicine.

[122] Wu CH, Guan MQ, Lu WH, Jiang XG, Zhang PH (2024). Lymphatic vessels are necessary for cardiac function and inflammation resolution in sepsis-induced cardiomyopathy. International immunopharmacology.

[123] Parmar D, Apte M (2021). Angiopoietin inhibitors: A review on targeting tumor angiogenesis. European journal of pharmacology.

[124] Becker F, Potepalov S, Shehzahdi R, Bernas M, Witte M, Abreo F (2015). Downregulation of FoxC2 Increased Susceptibility to Experimental Colitis: Influence of Lymphatic Drainage Function?. Inflammatory bowel diseases.

[125] Xu X, Greenland J, Baluk P, Adams A, Bose O, McDonald DM (2013). Cathepsin L protects mice from mycoplasmal infection and is essential for airway lymphangiogenesis. American journal of respiratory cell and molecular biology.

